# Supplementary material for: The PPARβ/Delta‐Induced Mesenchymal Stromal Cell Secretome Has Cytoprotective Effects via ANGPTL4 in a Pre‐Clinical Model of Acute Lung Inflammation
Source: FASEB J. 2026 Apr 20;40:e71820. doi: 10.1096/fj.202504659R (PMC13094458; doi:10.1096/fj.202504659R)
Supplement: Supplementary file 1 — Data S1: fsb271820‐sup‐0001‐Supinfo.pdf. [file FSB2-40-e71820-s001.pdf]

Supplementary Files

The PPARβ/delta-induced mesenchymal stromal cell secretome has cytoprotective effects via ANGPTL4 in a pre-clinical model of acute lung inflammation

Courteney Tunstead[1], Molly Dunlop[1], Sinéad Ryan[1], Evelina Volkova[1], Evangeline Johnston[1], Sabrina Batah[2], Claudia C. Dos Santos[2], Bairbre McNicholas [3,4], Claire Masterson[3,4], John G. Laffey[3,4] & Karen English[1,5,*]

Gene name	Brand	Species	Forward Sequence	Reverse Sequence
hprt	Sigma™	Human	5’ATAAGCCAGACTTTGTTGG	5’ATAGGACTCCAGATGTTTCC
top2a	Sigma™	Human	5'TCACAAGCAAGAAATCCAAG	5'ATCTTCATCTGACTCTTCCAG
angptl4	Sigma™	Human	5'AGGCAGAGTGGACTATTTG	5'CCTCCATCTGAGGTCATC
pdk4	Sigma™	Human	5'CTTGGGAAAAGAAGACCTTAC	5'GTGCAGTGGAGTATGTATAAC
ptgs2	Sigma™	Human	5'AAGCAGGCTAATACTGATAGG	5'TGTTGAAAAGTAGTTCTGGG
il-6	Sigma™	Human	5'GCAGAAAAAGGCAAAGAATC	5'CTACATTTGCCGAAGAGC
ido	Sigma™	Human	5'TTGTTCTCATTTCGTGATGG	5'TACTTTGATTGCAGAAGCAG
ptges	Sigma™	Human	5'CAAAAACATCACTCCCTCTC	5'AAAAGTCTGCATTCTTAGCC
tgfβ	Sigma™	Human	5'CCCACAACGAAATCTATGAC	5'TGTATTTCTGGTACAGCTCC

Supplementary table 1 (PRIMER SEQUENCES)

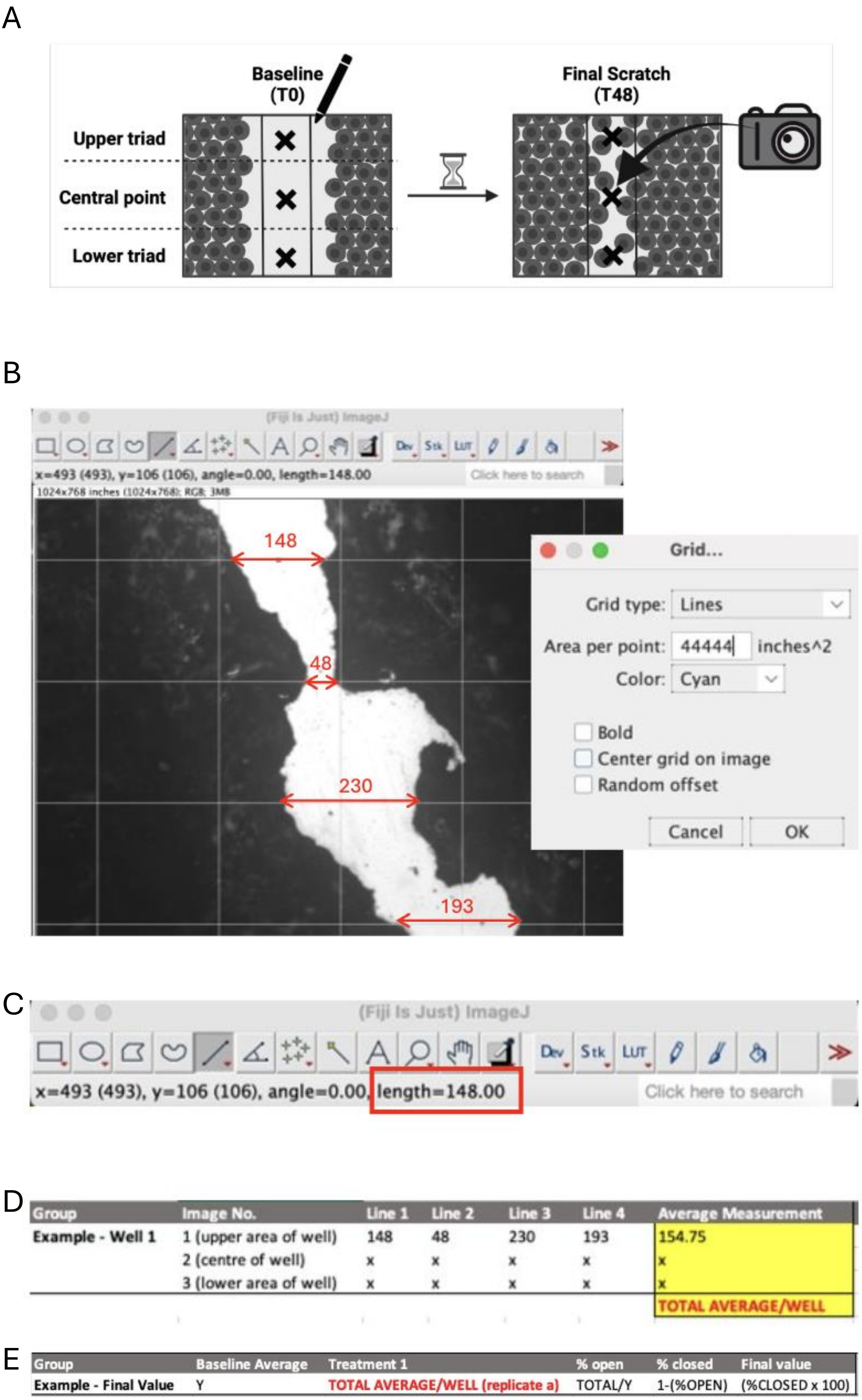

Group	Image No.	Line 1	Line 2	Line 3	Line 4	Average Measurement
Example - Well 1	1 (upper area of well)	148	48	230	193	154.75
	2 (centre of well)	x	x	x	x	x
	3 (lower area of well)	x	x	x	x	x
						TOTAL AVERAGE/WELL

Group	Baseline Average	Treatment 1	% open	% closed	Final value
Example - Final Value	Y	TOTAL AVERAGE/WELL (replicate a)	TOTAL/Y	1-(%OPEN)	(%CLOSED x 100)

Supplementary figure 1: Scratch assay analysis. CALU-3s were seeded at a density of 5x10[5] and allowed to grow until confluent. A single scratch was made per well, and images were taken from a region in the upper triad, central point and lower triad before analysing with ImageJ/Fiji software.

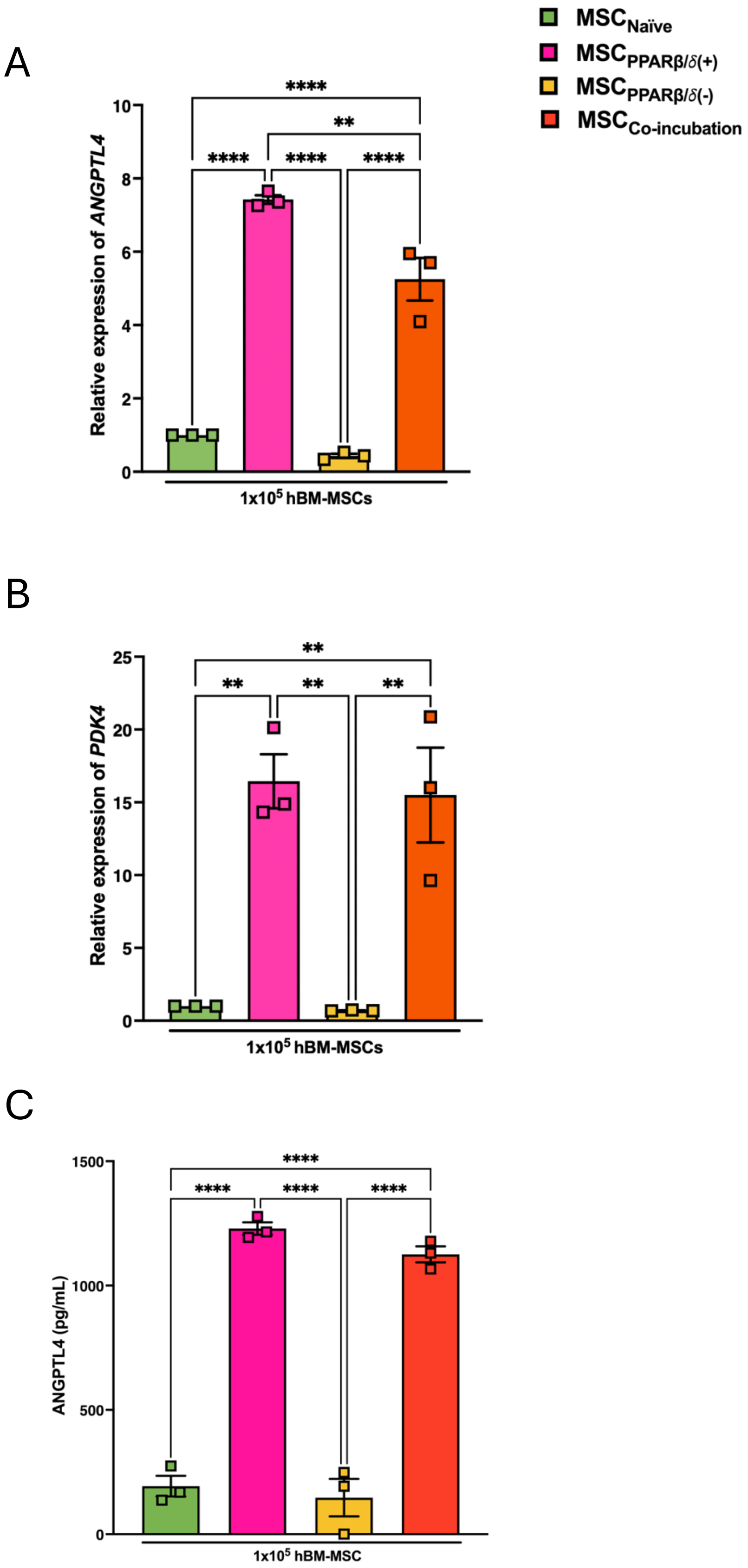

Supplementary figure 2: Co-incubation of PPARβ/δ-agonised and antagonised hBM-MSCs. hBM-MSCs were exposed to 1uM of a PPARβ/**δ** agonist *and* antagonist for 24hrs. Gene expression was assessed for (A) *angptl4* and (B) *pdk4* and protein expression was assessed for (C) ANGPTL4 (one-way ANOVA followed by Tukey's post-hoc test, n=3+). Replicates are a representation of individual hBM-MSC donors, with A and B previously presented in figure 2. Data is presented as mean ± SEM; **p<0.01, ***p<0.001, ****p<0.0001.

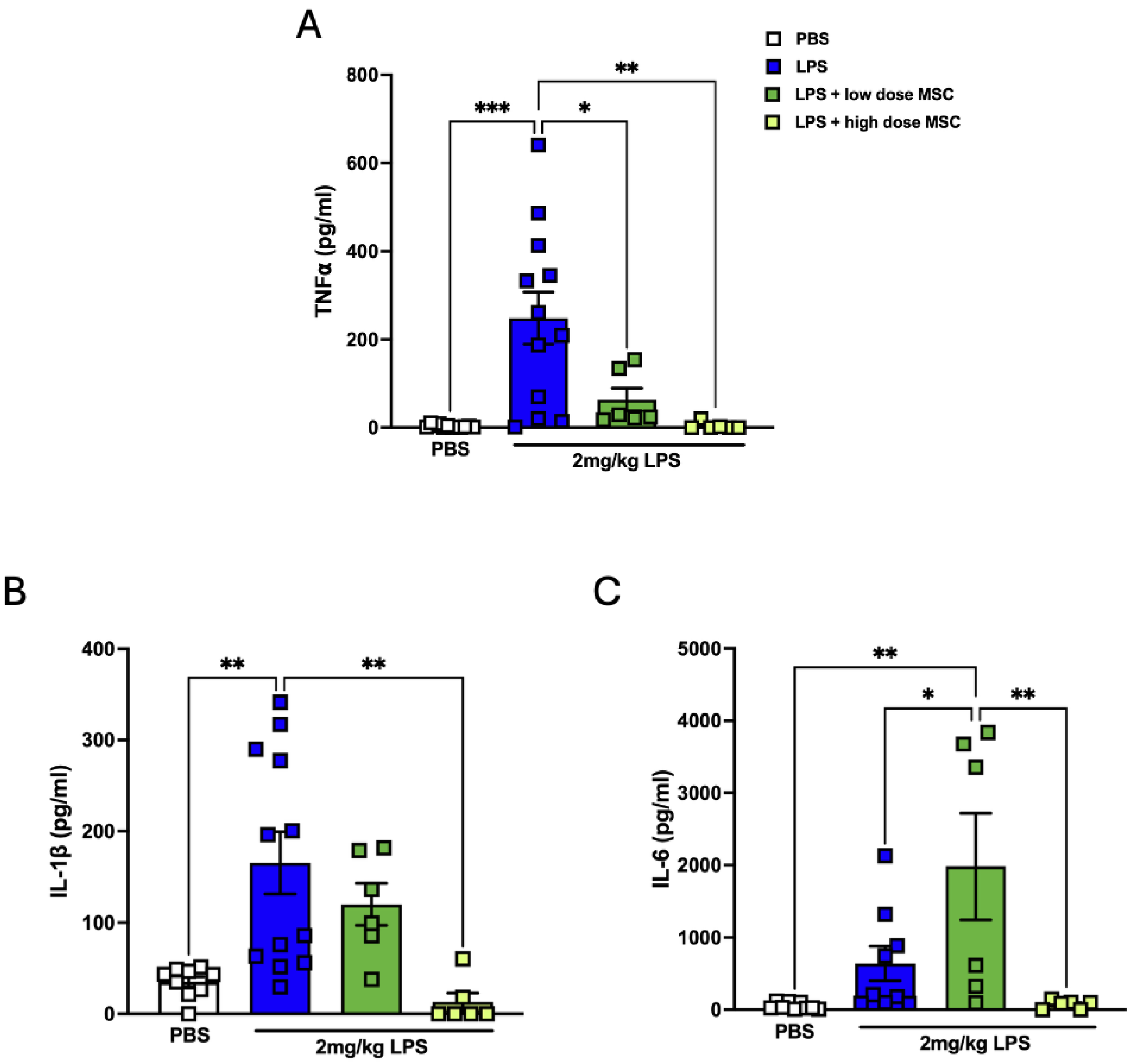

Supplementary figure 3: ALI mouse model pilot using intratracheal administration and dose curve of hBM-MSCs. Mice were exposed to 2mg/kg of LPS, or a PBS control, intratracheally and observed over 48hrs. They were further subjected to the administration of a low dose ($5x10^4$) or high dose ($1x10^5$) hBM-MSCs, intravenously. BALF pro-inflammatory cytokines (A) TNFα, (B) IL-1β and (C) IL-6 were measured by ELISA (one-way ANOVA followed by Tukey's post-hoc test, n=5-12). Replicates are a representation of individual mice. Data is presented as mean ± SEM; *p<0.05, **p<0.01, ***p<0.001.

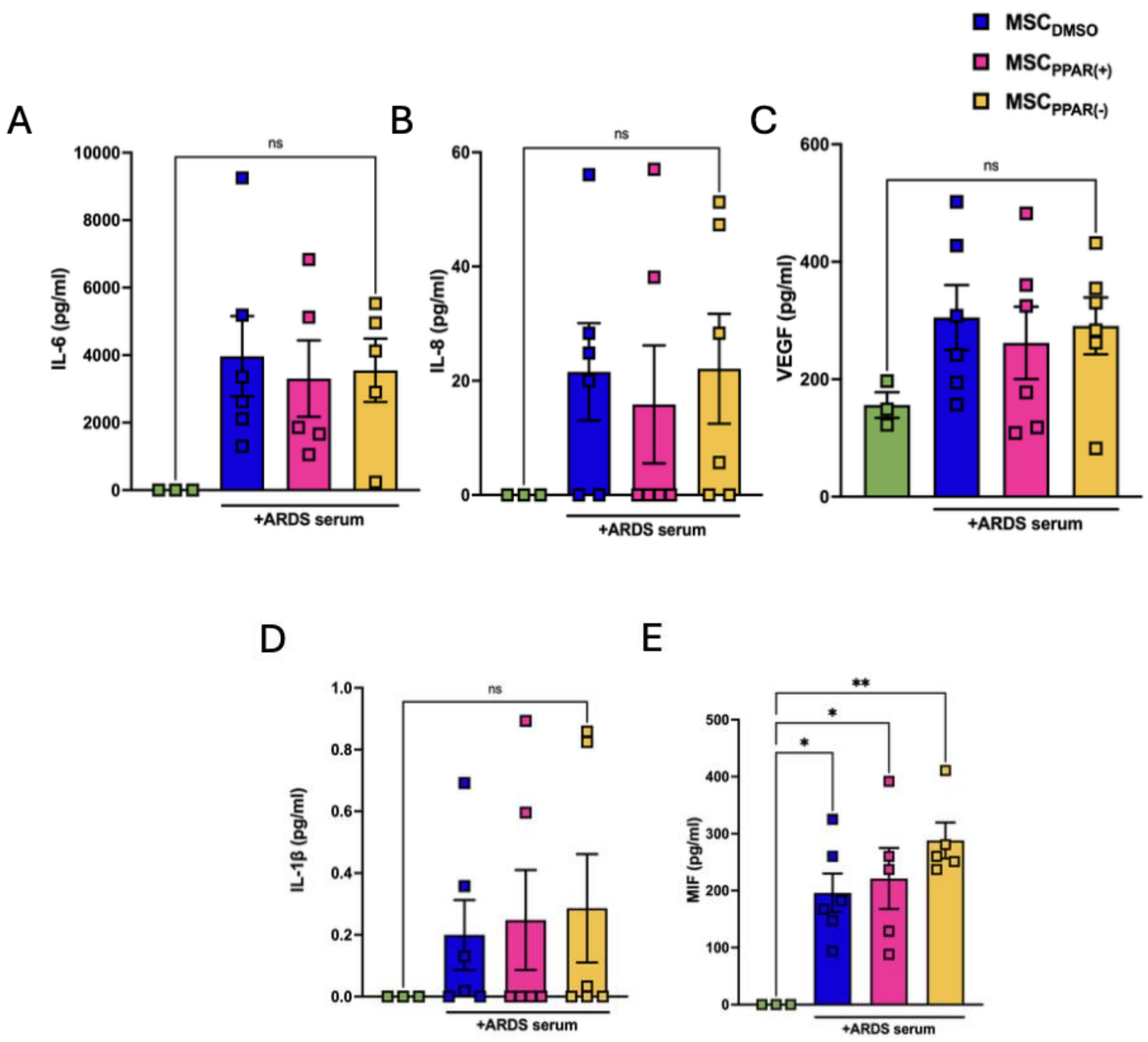

Supplementary figure 4: PPARβ/δ(+/-) hBM-MSCs in response to ARDS patient serum. hBM-MSCs were exposed to 1uM of a PPARβ/**δ** agonist or antagonist, and further exposed to ARDS patient serum. Protein expression was assessed for (A) IL-6, (B) IL-8, (C) VEGF, (D) IL-1β, (E) MIF (one-way ANOVA followed by Tukey's post-hoc test, n=3+). Replicates are a representation of individual hBM-MSC donors. Data is presented as mean ± SEM; *p<0.05, **p<0.01, ***p<0.001, ****p<0.0001.
